# The impact of transforming care on the care and safety of patients with intellectual disabilities and forensic needs

**DOI:** 10.1192/pb.bp.116.055095

**Published:** 2017-08

**Authors:** John L. Taylor, Iain McKinnon, Ian Thorpe, Bruce T. Gillmer

**Affiliations:** 1 Northumbria University, UK; 2 Newcastle University, UK; 3 Northumberland, Tyne and Wear NHS Foundation Trust, UK

## Abstract

NHS England recently published a national plan to develop community services for people with intellectual disabilities and autism who display challenging behaviour by using resources from the closure of a large number of hospital beds. An ambitious timescale has been set to implement this plan. The bed closure programme is moving ahead rapidly, but there has been little progress in developing community services to support it. This paper discusses the impact of the gap between policy and practice on the care and safety of patients with intellectual disabilities and forensic needs who form a distinct subgroup of the target population and are being disproportionately affected by this government policy.

## The policy context

*Building the Right Support,* a national plan to develop community services and close hospital beds for people with intellectual disabilities and autism who ‘display behaviour that challenges’ (p. 4), was published last October by NHS England and its local authority partners.^1^ The genesis of this plan was the Winterbourne View scandal in 2011, which involved the systematic abuse of people with intellectual disabilities in an independent sector hospital unit run by Castlebeck Care in Bristol, England.^2^ This led to a concordat that committed the Department of Health to a rapid reduction in the number of people with intellectual disabilities and challenging behaviour in hospital beds by mid-2014.^3^ Under pressure from politicians and stakeholder groups, who claimed that the government had failed to deliver on its concordat promises and that the situation had worsened,^4^ the chief executive of the National Health Service (NHS) committed to a 2-year intellectual disability hospital closure programme during a parliamentary select committee hearing on services for people with intellectual disabilities and challenging behaviour in early 2015.^5^

The ensuing national plan includes the closure of 45–65% of local clinical commissioning group (CCG)-commissioned and 25–40% of NHS England national specialist-commissioned in-patient beds by 2018. This is a ‘starting point’ and commissioners are encouraged to be ‘ambitious in thinking about how much further they can go’ (p. 6; unless stated otherwise, all quotations in this commentary are from *Building the Right Support* document^1^). The rationale for these numbers and timescale is unclear beyond what NHS commissioners and local authorities have told the plan's authors ‘they believe is possible’, which was then ‘sense-checked’ against geographical variations in current in-patient service usage (p. 27). The money saved from these bed closures is to be reinvested in the development of community services (p. 6). The national plan is clear that this transition will involve significant costs. It is stated that commissioners ‘will need to invest in new community support *before* closing inpatient provision’ (p. 7, italics added). Also required is the ‘temporary double running of services as inpatient facilities continue to be funded whilst new community services are established’ (p. 44). Local Transforming Care partnerships were tasked with drawing up implementation plans to support a new model of care and to start delivering against these plans by 1 April 2016.

## Lack of investment in community services

Unfortunately, little progress appears to have been made in agreeing, let alone implementing, new community service models to support the bed closure plans as envisaged in the national plan. The current authors work clinically with patients with intellectual and developmental disabilities who display offending and offending-type behaviours in in-patient and community services across six CCG areas in the north-east of England. As a group we have been closely involved in initiatives to increase hospital discharge rates and reduce readmissions, bed numbers and lengths of stay as well as to support and strengthen community services for people with intellectual disabilities who are at risk of offending. These innovations pre-date the Transforming Care programme and have already led to the closure of two in-patient units and a number of satellite beds in our services (40 beds in total). Despite our engagement and commitment to this transformation process we are concerned about the impact of the national plan, and the manner in which it is being implemented locally on patient care, patient safety and the safety of others.

The North East and Cumbria is one of six ‘fast-track areas’ in the national plan, set up with £2.06 million support from the NHS England Transforming Care programme to ‘help fund transitional costs and speed up implementation’ (p. 12). Fast-track areas aim to reduce in-patient bed usage by around 50% within 3 years, thereby ‘freeing up tens of millions of pounds which will be invested in community-based support to prevent hospital admissions’ (p. 13). The North East and Cumbria service model, which aims to deliver a 50% reduction in in-patient admissions, is currently in draft form and the ‘new community model’ embedded within the overall service model is not due to be considered by the North East and Cumbria Transforming Care Board until September 2016 at the earliest. Once the model is agreed, implementation plans will need to be developed, and resources including people and funds will need to be identified to enable it to be initiated. Judging by the pace of progress to date, this is likely to take considerable time. In the meantime, plans for in-patient bed closures are progressing rapidly, with 31 out of 112 beds (35%) across our medium- and low-secure and locked rehabilitation services currently empty as part of the closure programme.

## The effects of Transforming Care

The impact on patient care and safety of the drive to close in-patient beds without first having developed or strengthened community services is already beginning to show locally. The population served by these in-patient services, in contrast to the intended target population, is relatively high functioning intellectually (that is, mild/borderline in intellectual disability terms),^6^ shows high levels of psychiatric comorbidity^7^ and personality disorder characteristics,^8^ and generally does not display ‘behaviour that challenges’, but outwardly directed high-impact offending behaviour that has resulted in criminal convictions and/or detention under the Mental Health Act 1983 on the basis of ‘abnormally aggressive’ and/or ‘seriously irresponsible’ behaviour. Chief among the behaviours that bring these patients into these services are serious violence and aggression, sex offences, damage to property and firesetting.^9^ The most recent national census data reflect this offending behaviour profile, in that 33% of patients with intellectual disabilities detained under the Mental Health Act in England are subject to Part III criminal sections, and 21% of that group are subject to Ministry of Justice restrictions, meaning that they cannot be discharged without the approval of the Secretary of State or a mental health tribunal.^10^ Just 17% of in-patients with intellectual disabilities in the census were informal – that is, not detained under the Act.

The imperative to empty and then close in-patient beds has resulted in pressure being applied on clinical teams through commissioner-led ‘care and treatment reviews’^11^ to provide discharge dates for some forensic patients who continue to present levels of risk that local service providers and community teams are not adequately resourced to manage, or to consider transfers from NHS to independent hospital beds. Some evidence for the movement of patients around the in-patient system – possibly to create the illusion of progress – comes from a recent update from the NHS England Director of Transformation – Learning Disabilities,^12^ who reported that in April 2016, 20 of the net 100 recorded discharges were in fact transfers to other hospitals, and the destination of a further 20 discharged patients was unknown.

There is also concern that owing to the pressure to discharge as quickly as possible to meet the national plan targets, patients' rehabilitation is being hurried and/or truncated, resulting in some people being discharged before they are ready to take on the challenges of living in the wider community, or without the receiving community services being properly prepared to manage the risks these patients continue to present. The high level of clinical complexity and associated forensic risk in this population can require a significant period of assessment, formulation and specialist treatment to help patients develop thinking styles and attitudes, emotional control strategies and lifestyles less compatible with offending behaviour. A carefully considered and planned period of pre-discharge preparation is an important component of the treatment pathway and is essential to facilitating a successful transition from hospital to community care.

Another consequence of the current rapid bed closure policy is that people with intellectual disabilities and forensic needs who require urgent hospital treatment are being admitted to generic psychiatric services. This includes patients who have been previously detained in hospital under the Mental Health Act 1983 and discharged on community treatment orders, and who have been subsequently formally recalled to hospital owing to escalating risks of harm to themselves or others. Admission to acute psychiatry units can result in these patients being targeted and exploited by more able patients. In addition, they are unable to access appropriate assessment and treatment as the staff teams in these services have little or no experience of working with this population and lack the specialist skills required.^13^ This will result in longer periods of in-patient admission for these patients as access to suitable interventions aimed at reducing forensic risks is delayed.

One aim of the Transforming Care programme is to prevent people with intellectual disabilities and challenging behaviours from undergoing unnecessary admissions to intellectual disability and mental health in-patient services. Whether an admission is necessary or not is inevitably a matter of judgement. With the requirement for commissioner agreement to admission, there is a real risk of the judgement of clinicians being circumvented. Efforts to prevent admission to hospital by increasing supervision and support to people in community settings to manage emerging risks have paradoxically resulted in situations amounting to *de facto* deprivation of liberty in some cases, where a short informal hospital admission to allow the risks to be assessed and required amendments made to care plans would have been a less restrictive and more clinically effective option.

## Discussion and conclusions

People with intellectual disabilities who require treatment in hospital for behavioural, psychiatric and forensic problems should have access to the best evidence-based interventions available, delivered by caring staff with positive attitudes and person-centred values, in good-quality, safe environments. It is clear that a disproportionate number of people with intellectual disabilities are detained in hospital under the Mental Health Act^14^ and, once detained, they have on average longer lengths of stay than detained patients who do not have intellectual disability.^15^ The *Building the Right Support* national plan aims to address these inequities, albeit based on uncertain evidence and questionable assumptions.

There is no credible evidence or analysis presented to support the proposed bed reduction numbers. Between 1988 and 2015 the number of intellectual disability beds in the NHS reduced dramatically, from approximately 33 000 to about 2500.^1^ It is debatable whether this 90%-plus reduction over the past 30 years has been caused by centrally driven government policy initiatives, for example *Valuing People*,^16^ or the impact of human services theories, such as social role valorisation,^17^ on the deinstitutionalisation and community care movements in the 1980s and 90s.^18^ Either way, looking at the most recent census of in-patient services for people with intellectual disabilities in England,^10^ 83% were legally detained under the Mental Health Act 1983, with all of the scrutiny and protections this affords via mental health tribunals, hospital managers' hearings and Care Quality Commission inspections. It could be argued therefore that the majority of the remaining intellectual disability in-patient beds represent equipoise in the system and, as such, the current huge diversion of resources into forcing the closure of these remaining beds is unlikely to be successful in the long term.

The national plan starts from the supposition that all people with intellectual disabilities ‘should have a home within their community’ (p. 4). Seemingly underpinning this position is a belief that families and the community are always better for people with intellectual disabilities and that hospital services do not provide safety and sanctuary for some people. For many of our patients with forensic needs, their histories indicate that families and the community can be part of the problem rather than the solution. Putting to one side the fact that communities are generally not keen to embrace people who might have violently or sexually assaulted people in their midst, or set fire to their buildings, people with disabilities frequently experience abuse, aggression and violence in and by the community.^19^ There are many examples of people like Brent Martin, who was brutally murdered in 2007 by his more able ‘friends’ in Sunderland 3 months after being discharged from hospital.^20^

A further assumption underpinning the national plan is that hospital admissions should be as short as possible. There is an apparent lack of understanding that the population managed and treated by in-patient forensic intellectual disability services is distinct from the population envisaged within the Transforming Care programme. Patients with significant forensic histories have frequently experienced high levels of abuse, neglect and deprivation. They require time to develop insight into their difficulties in relating to others, acquire skills in regulating their emotions and acknowledge their future support needs. The application of a bed closure policy and as yet unclear community service model that is designed for a very different population carries significant risks of harm for patients with intellectual disabilities and forensic needs, as well as for others. The implementation of that policy without the required and promised investment in and development of community services is especially concerning. Some of the unintended consequences of this approach might include more vulnerable offenders with intellectual disabilities being sent to prison rather than diverted to hospital for appropriate treatment as recommended in the Bradley Report^21^ While imprisoned, such offenders will likely be targeted by other prisoners because of their disabilities and will remain at risk of re-offending, as they will be unable to access prison offending behaviour programmes ^22^ Finally, it is perhaps ironic that this policy will possibly lead to an increase in the use of independent sector hospital beds for people with intellectual disabilities – exactly where this all started.
